# Cardiac Efficiency

**Published:** 1920-04-03

**Authors:** 


### CARDIAC EFFICIENCY.

### Some Modern Methods of Examination.

The great prominence which requirements of war and recruiting gave to the limitations of most of our known cardiological methods is now a matter of history; but the lessons which were learned at the time, and those which, through many somewhat regrettable diagnostic inaccuracies, are now being forced upon the notice of those working on the various Ministry of Pensions boards, are not so well known among the general medical public. We hear it vaguely expressed that the bruit is no longer the keynote of cardiac diagnosis, and that the "exercise test" is the only one upon which reliance can be placed. And to some extent this is true, but a correct conception of our present state of knowledge can claim a considerably greater degree of refinement. In our contemporary, the American *Medical Record*, of February 21, we note a very valuable summary of the subject from the pen of Dr. T. E. Satterthwaite, and though it is impossible in a short note to cover more than a small proportion of the questions involved, yet it is worth while to select some of the more outstanding as being of exceptional value in the practice of everyday medicine among the public.

### The War Phase.

To be fair to all concerned in the earlier wartime work we must remember that time and press of circumstance would not allow more than a very brief physical examination, whereas later on investigations became more and more thorough, and the work became automatically allotted to those men presumably best fitted to carry it out. Also, the question in the first instance was whether a man was fit for service or not, and later it became whether a man who had developed apparent cardiac disability could be expected to return to active service. Obviously, as far as civil practice is concerned, these two aspects merge imperceptibly into each other, and methods employed in the post-service period are therefore of the greater immediate interest. Briefly may be recalled the so-called neurological tests. "Trigeminal irritation by the inhalation of strong smelling-salts that slow the normal heart but accelerate the neurotic. The oculo-cardiac, elicited by pressure on one or both eyeballs. By this method the normal rate is slowed four to ten beats per minute. Any increased degree of retardation suggests trouble with the cardioneural mechanism. Again, the administration of atropine, from $\frac{1}{25}$ to $\frac{1}{50}$ of a grain, increases the normal beat from thirty to forty per minute, while with hearts with myocardial degeneration the increase may be only twenty beats, or even less."

### Exercise Testing.

These tests may be classified as follows. Both the pulse and the systolic blood pressure are taken before and after the subject climbs a flight of twenty steps. Normally there is an increase of twenty beats per minute and a rise in pressure of eight mm. According to the degree of inefficiency, the rate rises higher and the pressure rises less, or perhaps actually falls. The length of time taken to return to normal is also important. Many modifications of this process have been devised and the best are perhaps those associated with uses of an ergo-meter, which actually measures the amount of muscular work done. Also, it is to be explained that in the *normal* heart under exercise, the systolic pressure rises after the acceleration of the rate, and after rest, is maintained longer than in the inefficient heart, where the pressure rise is both delayed and diminished.

Another method of changing the amount of working imposed upon the heart is to diminish the variation in range of circulating level by compressing both femoral arteries. As a matter of fact much the same thing applies to the test when a person changes from the recumbent to the sitting position. If the arterial pressure rises slightly when the load is taken off, the heart and the pulse rate does not rise or even slows a little, the heart is considered vigorous, and also if, when a load is suddenly thrown on, there is an increase in frequency with rapid reversion to the former rate in a normal heart, but in a degenerate heart there is no rapid compensatory change; the rate may, in fact, decrease a little.

Dr. Satterthwaite also refers his readers to several methods of comparing and estimating cardiac energy by simple calculations, after estimating the systolic and diastolic pressures, and the pulse rate, but straightforward though they are, we feel that these procedures are more adapted to the organised examination of Service men, factory operators, etc., rather than the average private patient. The same thing, indeed, applies to the physiological injection of atropine and adrenalin, so that, on the whole, the simple postural tests with blood pressure and pulse variations are likely to give most satisfaction to the practising general physician.

### Points of Diagnostic Interest.

Attention is called to the fact that in the now common use of mercurial manometers, oscillation of the column of mercury can give valuable indication of the type of pulse present, particularly in cases where digital impression is not readily received. Also the importance of taking blood pressures in both arms is noted. Particularly may a difference be observed in arterio-sclerotic condition. "Fortunately . . . . there has been a tendency of late to emphasise the fact that hypertension is misunderstood. Yet it is true that regardless of its causes it has an unfavourable outlook. It suggests renal difficulty or arterio-sclerosis, but may be due to a neurosis; to

**Cardiac Efficiency—(*continued*).**

excess in eating or drinking; to an idiosyncrasy; or to a familial peculiarity. Besides, it may be physiological. The first examination may show high pressure, the second a notable decrease in the pressure. These facts should be constantly borne in mind in making a prognosis. And yet, as I have intimated, high pressure must always be taken seriously. It does not, as a rule, suggest longevity, but there is every reason not to alarm the patient in any case. As these facts have not been generally known, the unfortunate patient's condition has sometimes been jeopardised from fear of a stroke, perhaps when there is really insufficient cause for alarm.''

On the subject of irritable, or '' soldier's heart,'' we have ourselves given a brief summary of the more modern conceptions (Aug. 30, 1919, p. 537), but it is interesting to read of the references obviously made to this very condition which came repeatedly under the physician's notice during the American Civil War. Sir James Barr and many others have elaborated upon the probability that it is in truth a condition of thyroidism and hyperactivity of the opposing nerve fibres in the autonomic system. We should, however, remember that there is a definite number of these cases, frequent in war conditions but also present in civilian life, where the origin is undoubtedly chronic absorption from some type of microbic infection. In the paper under notice, the author reviews Meakins' opinion that ''irritable'' heart may arise from a number of curious anomalies, concluding with considerations of psychoneuroses, blood-cell changes and asthenia, the whole being well summed up in saying that the state may be '' a conglomerate of functional disturbances rather than a pathological entity.'' Thus it cannot be too strongly impressed that although the heart symptoms and heart signs may provide the main points of light and shade in the clinical picture, yet the physician must view his case in the broadest manner and realise that the cardiac conditions may be but the mirror of other deep-seated disorders and, as so often occurs, great care is necessary to avoid treating the symptoms instead of the cause.

### Infective Cardiac Lesions.

Of the typical infective cardiac conditions one can at least say that they are perhaps among the best understood. At least we may accept the idea that in conditions such as rheumatism, scarlet fever, measles, etc., these different affections may have associated with them an endocarditis which is a direct sequence in the disease. But as Dr. Satterthwaite emphasises, '' We have also constantly before us claims that the meningococcus of cerebrospinal fever, the bacillus of influenza, and the *Streptococcus viridans* of oral sepsis are causes of endocarditis. But we are confronted with the statements of Rosenow, if he has been correctly quoted, that a streptococcus of the mouth or tonsils may change into the germ of pneumonia and back again to a micrococcus; and that a simple innocent variety can be converted by culture into an infecting agent, morphologically different and capable of producing many different diseases.'' On so extreme a claim as that quoted it is best to reserve our judgment, but it would appear fully probable that a variety of infective and other conditions may lower the bodily resistance and produce a myocarditis, and the low-grade streptococcal infection of the endocardium occur as a strictly secondary condition; in which case the elaborate metamorphoses of Rosenow are unnecessary to the completing of the story. The author himself cites several instances of this secondary type.

